# Periodontal Parameters in Fixed Labial and Lingual Orthodontic Treatment: A Systematic Review and Meta-Analysis

**DOI:** 10.3290/j.ohpd.b3601703

**Published:** 2022-11-23

**Authors:** Mingrui Zhai, Mengqiao Wang, Lan Li, Bohui Liu, Fulan Wei

**Affiliations:** a Postgraduate Student, Department of Orthodontics, School and Hospital of Stomatology, Cheeloo College of Medicine, Shandong University, Jinan, China; Shandong Key Laboratory of Oral Tissue Regeneration, Jinan, China; Shandong Engineering Laboratory for Dental Materials and Oral Tissue Regeneration, Jinan, China. Study concept, design and planning, collected data, wrote the manuscript, read and approved the final manuscript.; b Postgraduate Student, Department of Orthodontics, School and Hospital of Stomatology, Cheeloo College of Medicine, Shandong University, Jinan, China; Shandong Key Laboratory of Oral Tissue Regeneration, Jinan, China; Shandong Engineering Laboratory for Dental Materials and Oral Tissue Regeneration, Jinan, China. Study concept, design and planning, collected data, read and approved the final manuscript.; c Postgraduate Student, Department of Orthodontics, School and Hospital of Stomatology, Cheeloo College of Medicine, Shandong University, Jinan, China; Shandong Key Laboratory of Oral Tissue Regeneration, Jinan, China; Shandong Engineering Laboratory for Dental Materials and Oral Tissue Regeneration, Jinan, China. Study concept, design and planning, collected data, read and approved the final manuscript.; d Postgraduate Student, Department of Orthodontics, School and Hospital of Stomatology, Cheeloo College of Medicine, Shandong University, Jinan, China; Shandong Key Laboratory of Oral Tissue Regeneration, Jinan, China; Shandong Engineering Laboratory for Dental Materials and Oral Tissue Regeneration, Jinan, China. Study concept, design and planning, read and approved the final manuscript.; e Professor, Department of Orthodontics, School and Hospital of Stomatology, Cheeloo College of Medicine, Shandong University, Jinan, China; Shandong Key Laboratory of Oral Tissue Regeneration, Jinan, China; Shandong Engineering Laboratory for Dental Materials and Oral Tissue Regeneration, Jinan, China. Study concept, design and planning, collected and analysed data, wrote the manuscript, read and approved the final manuscript, project administration.

**Keywords:** meta-analysis, periodontal health, orthodontics, systematic review

## Abstract

**Purpose:**

To critically evaluate the periodontal parameters of patients receiving fixed labial and lingual orthodontic therapy.

**Materials and Methods:**

The current systematic review was registered at PROSPERO. Clinical studies comparing the periodontal parameters between fixed labial and lingual orthodontic treatment were searched up to June 2022 in four electronic databases, and unpublished literature was searched at ClinicalTrial.gov. The risk of bias of randomised controlled clinical trials (RCTs) and non-randomised clinical trials (n-RCTs) was assessed using the Cochrane risk-of-bias tool 2.0 and the Risk of Bias in Non-randomised Studies of Interventions (ROBINS-I) assessment tool, respectively. The pooled periodontal parameters were calculated in random-effect meta-analyses. The confidence of evidence was assessed via the Grading of Recommendations Assessment, Development and Evaluation (GRADE) approach.

**Results:**

Eight studies involving 223 patients were included in the current study. The risk of bias was high for 2 RCTs and 3 n-RCTs, and moderate for 3 n-RCTs. Patients receiving fixed lingual orthodontic treatment showed a lower plaque index (MD = -0.14; 95%CI -0.27 to -0.02). No statistically significant difference was found in the bleeding on probing index (MD = 0.11; 95%CI -0.03 to 0.25), gingival index (MD = 0.02; 95%CI -0.06 to 0.11), and periodontal pocket depth ( MD = 0.06; 95%CI -0.16 to 0.27) between the two groups. The overall quality of the evidence was very low to low.

**Conclusion:**

The present study indicates no obvious difference in periodontal parameters between the fixed labial and lingual orthodontic systems, although the overall quality was very low to low. Further RCTs with standardised outcome measures are needed.

**Supplement 1:** Reported outcomes of the included studies

Author, year	Outcome assessed	Measure of outcome/method of outcome assessment	Results	Conclusions
Tapia-Rivera et al, 2015	The presence of visible plaque Bleeding on probing Modified gingival index Simplified oral hygiene index	OR of following outcomes: Presence of plaque Bleeding on probing Modified gingival index Simplified oral hygiene index Method: Buccal surface of 11 and 31, lingual surface of 11 and 31, buccal surface of 16 and 26 Buccal and lingual surfaces of maxillary posterior teeth, buccal and lingual surfaces of mandibular posterior teeth Buccal surface of 11 and 31, lingual surface of 11 and 31, buccal surface of 16 and 26 Buccal surface of 11 and 31, lingual surface of 11 and 31.	Higher risk of presence of plaque on the buccal surface in the labial group, but higher risk of presence of plaque on the lingual surface in the lingual group. Higher risk of bleeding on probing on the buccal surface in the labial group, but risk of bleeding on probing on the lingual surface in the lingual group. Higher risk of modified gingival index on the buccal surface in the labial group, but risk of modified gingival index on the lingual surface in the lingual group. No statistically significant difference in simplified oral hygiene index between the labial and lingual groups.	The clinical periodontal health conditions may be considered acceptable for patients using both conventional and lingual brackets.
Bruno et al, 2019	Plaque index Gingival index	Mean ± SD of following outcomes: Plaque index at the vestibular, lingual, mesial and distal surface. Gingival index at the vestibular, lingual, mesial and distal surface. Measurements of all teeth	No statistically significant difference in plaque index between the labial and lingual groups. No statistically significant difference in gingival index between the labial and lingual groups.	There is no statistically significant difference between the vestibular and lingual appliances.
Gujar et al, 2020	Plaque index Gingival index Bleeding on probing index	Not reported	No statistically significant difference in plaque index between the labial and lingual group. No statistically significant difference in gingival index between the labial and lingual groups. No statistically significant difference in bleeding on probing index between the labial and lingual groups.	No statistically significant difference with regard to plaque index, gingival index, and bleeding on probing index between the labial and lingual groups.
Lombardo et al, 2013	Decayed, missing, and filled teeth index Plaque index Gingival bleeding index Salivary flow rate Salivary buffer capacity and pH Streptococcus mutans count Lactobacillus count	Mean ± SD in decayed, missing, and filled teeth index Plaque index were expressed as percentages of the total number of tooth surfaces examined Gingival bleeding index were expressed as percentages of the total number of tooth surfaces examined Mean ± SD in salivary flow rate Mean ± SD in salivary buffer capacity and pH. Defined the counts into low, moderate or high level according to the colony counts. Described as percentage of low, moderate or high level. All teeth At six sites around each tooth At six sites around each tooth Salivary collections for salivary flow rate, salivary buffering capacity and pH, *Streptococcus mutans* count, and *Lactobacillus* count.	No statistically significant difference was found in plaque index between the fixed labial and lingual groups. No statistically significant difference was found in gingival bleeding index between the fixed labial and lingual groups. No statistically significant difference was found in salivary flow rate between the fixed labial and lingual groups. No statistically significant difference was found in salivary buffer capacity and pH between the fixed labial and lingual groups. No comparison in *Streptococcus mutans* count and *Lactobacillus* count between the fixed labial and lingual groups.	Higher plaque deposited, more gingival inflammation and more S. mutans counts in the lingual group. No statistically significant difference was found in lactobacillus counts, the salivary flow rate, and saliva buffer capacity.
Sfondrini et al, 2012	Colony-forming units Periodontal pocket depth Bleeding on probing index	The total count of microorganisms was determined on countable plates. The pocket depths were measured at the buccal, lingual, mesial and distal sides of the tooth and rounded off to the nearest 0.5 mm. BOP was recorded (0: absent; 1: present) 24 s after determination of PPD. Measurement of the canine and the first premolar of each quadrant	No statistically significant differences were found between buccal and lingual brackets in terms of colony-forming units. No statistically significant differences were found between buccal and lingual brackets in terms of periodontal pocket depth. No statistically significant differences were found between buccal and lingual brackets in terms of bleeding on probing index.	Bracket position does not have a statistically significant impact on bacterial load or periodontal parameters.
Vijaykuma et al, 2020	Plaque index Calculus index Gingival index	Mean ± SD in plaque index. Mean ± SD in calculus index. Mean ± SD in gingival index. Method not reported.	In the third month, all three indices were statistically significant for both labial and lingual therapy. The lingual appliance showed more plaque and calculus accumulation.	The lingual surface of patients undergoing lingual orthodontic treatment exhibited more plaque and calculus deposition, thereby worsening the periodontal status.
Wang et al, 2018	Gingival index Plaque index Sulcular bleeding index Periodontal depth	Gingival tissues were divided into four areas for scoring: mesial, distal, buccal, and lingual area. Mean ± SD in gingival index. Mean ± SD in plaque index. Mean ± SD in sulcular bleeding index. Mean ± SD in periodontal depth. 16, 21, 24, 36, 41 and 44 were chosen for tooth detection	No statistical significance was observed throughout the changing pattern. No statistical significance was noted in the periodontal index between two groups after 1-, 3- and 6-month treatment	It is more difficult to maintain the oral hygiene after lingual orthodontics compared with labial orthodontics, whereas the status of periodontal health does not statistically significantly differ after lingual and labial orthodontic treatment.
Zhang et al, 2018	Gingival index Bleeding on probing index Periodontal pocket depth	Mean ± SD in gingival index. Mean ± SD in bleeding on probing index. Six sites around the teeth were detected. Mean ± SD in periodontal pocket depth. Measurements of 16, 11, 31, and 46	The PD, GI, and BI values were higher in both groups at the 3rd and 6th month of correction compared with those before correction. The PD, GI, and BI were statistically significantly higher in the lingual group than in the labial group at the 3rd and 6th month of correction. The number of bacteria other than *Actinobacillus actinomycetemcomitans* in the lingual group was higher than that in the labial group at months 3 and 6 of correction.	Compared with labial fixed orthodontic treatment, the effect of lingual fixed orthodontic treatment affects the cleanliness of periodontal tissues, and the maintenance of oral health should be a priority when lingual fixed orthodontic treatment is performed.

Mean: mean difference; SD: standard deviation.

Orthodontic treatment aims to provide an acceptable functional and aesthetic occlusion. Fixed orthodontic appliances have become an integral part of modern orthodontics and are extensively used in clinics.^17^ However, fixed orthodontic appliances have been reported to be associated with impaired oral hygiene because of the increased niches for plaque retention and biofilm formation, as well as the increased difficulty in removing plaque mechanically.^29,42^ It has been reported that once the homeostatic balance between subgingival microbial communities and adjacent host tissue has been disturbed, the accumulated subgingival biofilms would initiate the development of gingivitis.^6,16,18^ Furthermore, gingivitis can further progress to periodontitis, which may result in long-term, irreversible damage to periodontal tissues.^5,30^ Therefore, it is of critical importance to monitor the alterations in clinical periodontal parameters after fixed orthodontic appliance insertion.

Traditional fixed orthodontic appliances were fixed on the labial surface of the teeth. Recently, the fixed lingual orthodontic appliance was invented and developed, and has since become an alternative to conventional labial orthodontic appliances due to its excellent aesthetics and growing practicability.^8,27^ Numerous investigations have been conducted to compare traditional labial orthodontic treatment and novel lingual orthodontic treatment.^1,22,27,42^ Evidence has confirmed that satisfying treatment outcomes could both be achieved by using either of the two orthodontic appliances.^23^ When taking clinical periodontal parameters into account, the standpoints on comparison between the two fixed orthodontic systems have been controversial. Some found deteriorated oral hygiene and periodontal parameters during fixed lingual orthodontic treatment compared to traditional labial orthodontic therapy.^21,40,43^ In contrast, others reported a lower risk of caries in the fixed lingual orthodontic treatment. However, to our knowledge, the investigations concerning clinical periodontal parameters have never been pooled in a systematic way.

Several systematic reviews have made great efforts to compare oral hygiene between fixed labial and lingual orthodontic treatment,^1,22,27^ revealing greater difficulty in taking oral hygiene measures with fixed lingual brackets. However, these reviews only focused on oral hygiene and the difficult access during hygiene measures, instead of clinical periodontal parameters, which were more objective and were appraised by professional periodontists. The two available reviews regarding oral hygiene and the difficulty of performing hygiene measures were conducted almost six years ago.^1,27^ Since then, many primary studies have been published.^3,10,40,41,43^ No comprehensive approach has been followed to date to review the existing evidence on clinical periodontal parameters. Therefore, the aim of the present systematic review was to assess the current literature in terms of the comparison of periodontal parameters between patients receiving fixed labial and lingual orthodontic treatment.

## Materials and Methods

This meta-analysis was conducted and reported according to the Cochrane Handbook for Systematic Reviews of Interventions, version 6.2, and the 2020 checklist for the Preferred Reporting Items for Systematic Reviews and Meta-Analysis (PRISMA).^12,26^ The protocol for this systematic review and meta-analysis was registered with the International Prospective Register of Systematic Reviews (PROSPERO) (https://www.crd.york.ac.uk/PROSPERO/) under registration number CRD42020188538.

### Eligibility Criteria

According to the “participants, intervention, comparison, outcome, and study design (PICOS)” principle, the current study was designed to compare the clinical periodontal parameters between patients receiving fixed labial and lingual orthodontic treatment. The inclusion and exclusion criteria for study selection were:

- Patients: all patients treated with fixed labial and all patients treated with lingual orthodontic appliances were included; patients with systemic diseases, cleft lip, cleft palate, or other craniofacial abnormalities were excluded.
- Interventions: studies using fixed labial and lingual orthodontic appliances were included; additional treatments such as maxillofacial surgery were excluded.
- Comparison: fixed labial orthodontic treatment vs fixed lingual orthodontic treatment; excluded if there was no comparison between fixed labial and lingual orthodontic treatment.
- Outcome: all clinical periodontal parameters were included; outcomes not relevant to periodontal status were excluded.
- Study design: randomised controlled clinical trials and non-randomised clinical trials were included; systematic reviews, case reports, in-vitro studies, or animal studies were excluded.

### Information Sources and Literature Search

The following four electronic databases were searched up to June 2022 without any limits: Web of Science, Embase, Pubmed, and Scopus. In addition, studies from the grey literature were searched in ClinicalTrial.gov. Hand searching was conducted in the retrieved literature for any additional articles that were eligible for the current systematic review. The search strategies applied are detailed in Table 1.

**Table 1:** Search strategies applied in the current study

Database	Search methods
Web of Science	#1 TS= (buccal OR labial OR vestibular) OR TS= (lingual) #2 TS= (bracket* OR orthodontic) #3 TS= (vivo OR clinical) #4 TS= (vitro) #5 TS= (periodontal OR gingival OR bleed* OR adverse) #1 AND #2 AND #3 AND #5 NOT #4
Pubmed	((“buccal”[All Fields] OR “buccally”[All Fields] OR (“labially”[All Fields] OR “lip”[MeSH Terms] OR “lip”[All Fields] OR “labial”[All Fields] OR “labials”[All Fields]) OR “vestibular”[All Fields] OR (“lingualized”[All Fields] OR “lingually”[All Fields] OR “tongue”[MeSH Terms] OR “tongue”[All Fields] OR “lingual”[All Fields])) AND (“bracket”[All Fields] OR “bracket s”[All Fields] OR “brackets”[All Fields] OR (“orthodontal”[All Fields] OR “orthodontic”[All Fields] OR “orthodontical”[All Fields] OR “orthodontically”[All Fields] OR “orthodontics”[MeSH Terms] OR “orthodontics”[All Fields])) AND (“vivo”[All Fields] OR (“clinical trials as topic”[MeSH Terms] OR (“clinical”[All Fields] AND “trials”[All Fields] AND “topic”[All Fields]) OR “clinical trials as topic”[All Fields] OR “trial”[All Fields] OR “trial s”[All Fields] OR “trialed”[All Fields] OR “trialing”[All Fields] OR “trials”[All Fields])) AND (“periodontal”[All Fields] OR “periodontally”[All Fields] OR “periodontically”[All Fields] OR “periodontics”[MeSH Terms] OR “periodontics”[All Fields] OR “periodontic”[All Fields] OR “periodontitis”[MeSH Terms] OR “periodontitis”[All Fields] OR “periodontitides”[All Fields] OR (“gingiva”[MeSH Terms] OR “gingiva”[All Fields] OR “gingival”[All Fields] OR “gingivally”[All Fields] OR “gingivals”[All Fields] OR “gingivitis”[MeSH Terms] OR “gingivitis”[All Fields] OR “gingivitides”[All Fields]) OR (“bleedings”[All Fields] OR “hemorrhage”[MeSH Terms] OR “hemorrhage”[All Fields] OR “bleed”[All Fields] OR “bleeding”[All Fields] OR “bleeds”[All Fields]) OR (“adverse”[All Fields] OR “adversely”[All Fields] OR “adverses”[All Fields]))) NOT “vitro”[All Fields]
Scopus	(TITLE-ABS-KEY (buccal OR labial OR vestibular) OR TITLE-ABS-KEY (lingual) AND TITLE-ABS-KEY (bracket OR orthodontic) AND TITLE-ABS-KEY (vivo OR clincial) AND NOT TITLE-ABS-KEY (vitro) AND TITLE-ABS-KEY (periodontal OR gingival OR bleed OR adverse))
Embase	“(buccal OR labial OR vestibular) OR (lingual) AND (periodontal OR gingival OR bleed OR adverse) AND (vivo OR clincial) NOT (vitro)”
Clinicaltrial.gov	periodontal | orthodontic | fixed buccal and lingual orthodontic bracket

### Study Selection

All the studies retrieved from the search procedures were imported into Endnote software to discard duplicates. Two independent reviewers evaluated the eligibility of each study according to the pre-established inclusion and exclusion criteria. Briefly, the titles and abstracts of the studies were independently screened by two reviewers. The full text of the studies was obtained and assessed for fulfilling the inclusion criteria after screening the titles and abstracts. When necessary, corresponding authors were contacted for further information. Any disagreements concerning the eligibility were resolved by consensus; otherwise, the opinion of the third reviewer was referred to.

### Risk of Bias Assessment

The assessment of the risk of bias in randomised controlled clinical trials (RCTs) and non-randomised clinical trials (n-RCTs) was conducted according to the Revised Cochrane risk-of-bias tool 2.0 for randomised trials^35^ and the Risk Of Bias In Non-randomised Studies of Interventions (ROBINS-I) assessment tool.^34^

### Data Extraction

A pre-established data extraction table was used for information extraction; this procedure was also conducted by two independent reviewers. Missing data were acquired from the corresponding author of the pertinent literature; otherwise, only the available data was included.

### Data Analysis

Methodological and clinical heterogeneity were evaluated by examining the study characteristics, similarity of participant characteristics, interventions, and study outcomes, as specified in the inclusion and exclusion criteria for the present systematic review. The I^2^ and Χ^2^ tests were used to determine statistical heterogeneity.^13^ Meta-analysis was performed using Review Manager 5 (version 5.3; Nordic Cochrane Centre, Cochrane Collaboration; Copenhagen, Denmark). Mean differences (MD) and standard deviations (SD) were used to summarise the data from each study. Pooled estimates and 95% confidence intervals (CI) were presented if two or more studies reporting consistent periodontal indices were deemed eligible for a single comparison. Further, a random-effects model was applied in the current meta-analysis.^33^ Forest plots were made to graphically explain the meta-analysis results, in which the effect size of each outcome and the heterogeneity were presented. Subgroup analyses based on follow-up periods were pre-determined to explore the effect of the follow-up time on pooled results. Sensitivity analyses were also conducted, if applicable. The formal testing for publication bias was evaluated using funnel plots and the Egger test, if there were at least ten studies involved.^19^

### Quality Assessment of the Outcome

The overall quality of evidence was evaluated through the GRADE approach using GRADE profile version 3.6 software.^11^ When assessing the overall quality, the risk of bias of each outcome in each study was evaluated again, so that the risk of bias was assessed at the outcome level.

## Results

The data that support the findings of this study are available from the corresponding author upon reasonable request.

### Characteristics of Included Studies

**Fig 1:**
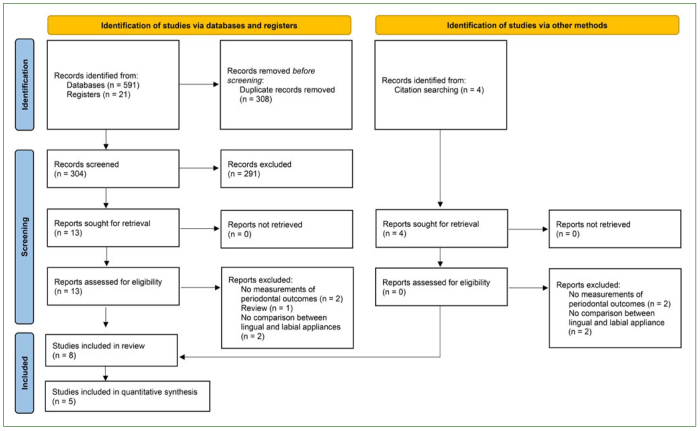
PRISMA flow diagram of the search processes and results.

The study flowchart is shown in Fig 1. A total of 616 papers were initially retrieved, and 308 studies were left after removing duplicates. Finally, 8 studies comprising 223 patients^3,10,21,31,36,40,41,43^ were considered eligible for the present systematic review, and 5 studies^3,31,40,41,43^ were appropriate for quantitative analyses. In terms of including and excluding studies, there was substantial agreement between the two reviewers (K = 0.973).^4^

Two of the included studies were RCTs^3,31^ and six were n-RCTs.^10,21,36,40,41,43^ Seven studies^3,10,21,36,40,41,43^ were based on a parallel two-arm design, and one study^31^ was based on a split-mouth design. The sample size of included studies ranged from 20 to 83 participants with age varying from 19 to 34 years. Balanced recruitment of participants of similar age was present in the majority of studies included in the current review. However, whether or not the distribution of gender was similar between the two groups could not be verified due to missing data in several investigations.^40,41^ The main reported clinical periodontal outcomes focused on four clinical periodontal parameters: plaque index (PI), gingival index (GI), bleeding on probing index (BOP), and periodontal pocket depth (PPD). The follow-up duration ranged from one week after the start of orthodontic treatment to one month after completing orthodontic treatment. Other basic data and information could be found in Table 2.

**Table 2:** Summary of characteristics of studies included in the systematic review

Author and year	Study Design	Sample Size	Age	Gender	Outcomes	Timepoints of measurements
Vijaykumar et al, 2020	Non-randomised clinical trial (two-arm, parallel)	Labial: N = 10 Lingual: N = 10	NR	NR	PI CI GI	T1: one month T2: three months
Zhang et al, 2018	Non-randomised clinical trial (Two-arm parallel)	Labial: N = 15 Lingual: N = 15	NR	Labial: M/F = 6:9 Lingual: M/F = 8:7	PPD GI BOP	T0: before treatment T1: three months T2: six months
Lombardo et al, 2013	Non-randomised clinical trial (Two-arm parallel)	Labial: N = 10 Lingual: N = 10	Labial: 19.3 ± 3.6 Lingual: 22.3 ± 3.2	Labial: M/F = 2:8 Lingual: M/F = 3:7	PI BOP Salivary flow rate Salivary pH Streptococcus mutans counts Lactobacillus counts	T0: before placement T1: 4 weeks T2: 8 weeks
Sfondrini et al, 2012	Randomised controlled trial (split-mouth)	Labial: N = 20 Lingual: N = 20	Labial: 23.8 ± 2.4 Lingual: 23.8 ± 2.4	Labial: M/F = 6:14 Lingual: M/F = 6:14	PPD BOP Streptococci count Anaerobe count Total count	T0: 1 day T1: 7 days T2: 30 days
Bruno et al, 2019	Randomised controlled trial (Two-arm parallel)	Labial: N = 10 Lingual: N = 10	NR	Labial: M/F = 2:8 Lingual: M/F = 1:9	PI GI	T0: before bonding T1: one month T2: three months T3: six months
Tapia-Rivea et al, 2015	Non-randomised clinical trial (Two-arm parallel)	Labial: N = 40 Lingual: N = 43	SP Labial: 19.6 ± 8.9 Lingual: 34 ± 12.1 MS Labial: 28.8 ± 14.2 Lingual: 29.2 ± 10.5	SP Labial: NR Lingual: M/F = 40% MS Labial: NR Lingual: M/F = 29%	VP BOP OHI-S MGI	At three months after bonding brackets
Wang et al, 2018	Non-randomised clinical trial (Two-arm parallel)	Labial: N = 16 Lingual: N = 14	NR	NR	GI PI PPD SBI	T0: before bracket placement T1: one month T2: three months T3: six months T4: one month after treatment

N: number; SP: São Paulo clinic; MS: Mato Grosso do Sul clinic. M/F: the ratio of male and female; NR: not reported; RCT: randomised controlled trial; CCT: controlled clinical trial; Labial: fixed labial orthodontic treatment group; lingual: fixed lingual orthodontic treatment group; PI: plaque index; CI: calculus index; GI: gingival index; PPD: periodontal pocket depth; BOP: bleeding on probing index; MGI: modified gingival index; OHI-S: oral hygiene index; VP: visible plaque; SBI: sulcus bleeding index.

### Risk of Bias of Included Studies

Two included RCTs^3,31^ were both evaluated as high risk of bias using the ROB2.0 assessment tool (Table 3). Considering randomisation, Sfondrini et al^31^ performed randomisation by means of concealed envelopes, and Bruno et al^3^ used a random numbers table of the groups. The high risk of bias mainly pertained to deviations from the intended interventions in both studies. Only Sfondrini et al^31^ claimed that the periodontists who were responsible for periodontal-parameter evaluation were blinded for previous scores; Bruno et al^3^ did not mention the measure for blinding evaluators. No missing outcome data or bias of selective report was found among the included RCTs.

**Table 3:** Risk of bias assessment according to Cochrane risk-of-bias tool 2.0

Study	Sfondrini et al, 2012	Bruno et al, 2019
Risk of bias arising from the randomisation process	Low	Low
Risk of bias due to deviations from the intended interventions	High	High
Missing outcome data	Low	Low
Risk of bias in measurement of the outcome	Some concerns	High
Risk of bias in selection of the reported result	Low	Low
Overall risk of bias	High	High

Among n-RCTs, three^21,41,43^ were considered to have moderate bias, while the other three^10,36,40^ were assessed as possessing serious bias according to the ROBINS-I assessment tool (Table 4). The most severely affected domains were the selection of participants and missing data. Bias in deviations from intended interventions as well as blindness in outcomes measurements could not be ignored. In terms of reporting bias and classification of interventions, all involved studies were considered as having a low risk of bias due to sufficient details provided, and reported and specific classification of interventions.

**Table 4:** Risk-of-bias assessment of non-randomised clinical trials using ROBINS-I tool

Domains	Vijaykumar et al, 2020	Zhang et al, 2018	Lombardo et al, 2013	Tapia-Rivera et al, 2015	Wang et al, 2018	Gujar et al, 2020
Bias due to confounding	Low	Low	Low	Serious	Low	Serious
Bias in selection of participants into the study	Serious	Low	Moderate	Serious	Moderate	Low
Bias in classification of interventions	Low	Low	Low	Low	Low	Low
Bias due to deviations from intended interventions	Low	Moderate	Low	Moderate	Low	Low
Bias due to missing data	Low	Low	Low	Serious	Low	Serious
Bias in measurement of outcomes	Low	Moderate	Low	Moderate	Low	Moderate
Bias in selection of reported results	Low	Low	Low	Low	Low	Low
Overall bias	Serious	Moderate	Moderate	Serious	Moderate	Serious

### Results of Individual Studies

The results and conclusions of individual studies are described in Supplement 1, in which we provide an overview of the difference in clinical periodontal parameters between fixed labial and lingual orthodontic therapy.

### Results of Meta-Analyses and Additional Analyses

Three studies^10,21,36^ were not included in the meta-analyses due to using different periodontal assessment indices with high clinical heterogeneity. Specifically, Lombardo et al^21^ reported the presence of PI and gingival bleeding index (GBI) with continuous statistics, the study of Gujar et al^10^ did not report which periodontal indices applied. Tapia-Rivera et al^36^ reported the presence of visible PI, modified GI (dichotomised as adequate and mild gingivitis), and the presence of BOP as dichotomised results.^36^ As the odds ratio (OR) was only reported in one study^36^ and these dichotomised results without raw data could not be pooled with continuous results, this study was not included in the current meta-analysis. Therefore, only 5 studies were involved in the quantitative analyses.

**Fig 2:**
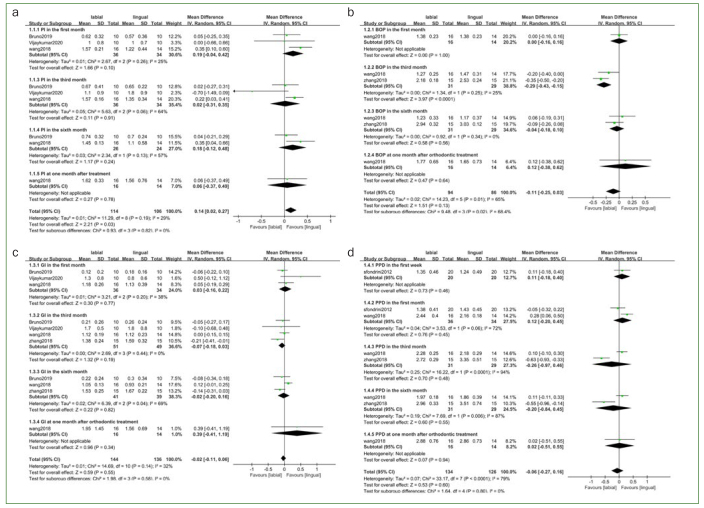
Forest plots for clinical periodontal parameters. a. plaque index; b. bleeding on probing index; c. gingival index; d. periodontal pocket depth.

Three investigations^3,40,41^ reported plaque index according to the Loe-Silness method,^32^ comprising 70 participants who were pooled statistically. In terms of tooth position detection, Wang et al^41^ chose Ramfjord teeth (tooth numbers 16, 21, 24, 36, 41, and 44) for tooth detection, while Bruno et al^3^ assessed all teeth and Vijaykumar et al^40^ only evaluated the PI of mandibular anterior teeth. However, only Bruno et al^3^ described examining four sites (vestibular, lingual, mesial, and distal surfaces) for PI, and the other two did not report specific sites per tooth chosen for measurement. Low statistical heterogeneity was observed across the related studies (Χ^2^ = 11.25; I^2^ = 29%). PI was statistically significantly lower in patients receiving the fixed lingual orthodontic treatment compared to those undergoing traditional fixed labial orthodontic treatment (MD = 0.14; 95% CI, 0.02 to 0.27), while subgroup analyses yielded no statistically significant difference at each follow-up timepoint (Fig 2a). With regard to BOP, two studies^41,43^ comprising 60 participants were included in the quantitative synthesis. Both studies evaluated BOP according to the Mazza method,^24^ yet the tooth positions detected were different and no specific sites per tooth were reported. As opposed to Wang et al,^41^ who chose Ramfjord teeth to assess BOP, Zhang et al^43^ only assessed the BOP of four teeth (tooth numbers 16, 11, 31, and 46), but neither reported specific sites chosen per tooth for measurement. We found no statistically significant difference in BOP between the labial and lingual group (MD = -0.11; 95% CI, -0.25 to 0.03), with moderate heterogeneity (Χ^2^ =14.23; I^2^ = 65%). Similarly, no statistically significant difference was found in terms of BOP at each follow-up timepoint according to subgroup analyses (Fig 2b). Regarding GI, four studies^3,40,41,43^ using the Loe-Silness method^20^ were deemed eligible for meta-analysis. Wang et al^41^ and Bruno et al^3^ took the GI at four sites per tooth, while Vijaykumar et al^40^ and Zhang et al^43^ did not report the specific site in detail. The meta-analysis in terms of GI involved 100 participants and these 100 participants were the sum of the participants in the four experiments described above. No statistically significant difference in GI between patients undergoing fixed lingual orthodontic therapy and those with traditional labial orthodontic appliances (MD = -0.02; 95% CI, -0.11 to 0.06; Χ^2^ = 14.69; I^2^ = 32%) was observed. Our results of subgroup analyses according to follow-up duration were consistent with the former results^3,41^ (Fig 2c). Three studies^31,41,43^ which reported PPD results from 80 participants were deemed eligible for quantitative analysis. Zhang et al^43^ reported the mean value of six sites around the measured teeth (tooth numbers 16, 11, 31 and 46), while the other two^31,41^ did not report tooth positions or specific sites at which PPD was measured. Similar PPDs were observed using the two systems, but considerable heterogeneity was found (MD = -0.06; 95% CI, -0.27 to 0.16; Χ^2^ = 33.17; I^2^ = 79%). Subgroup analyses showed no statistically significant difference between the two groups at each follow-up period (Fig 2d). Further sensitivity analysis or publication bias assessment were ultimately not conducted, due to the paucity of existing studies contributing to the quantitative synthesis.

### Risk of Bias Across the Studies

The overall quality of evidence assessed by the GRADE approach is exhibited in Table 5. As recommended in the Cochrane Handbook for Systematic Reviews of Interventions Version 6.2, the overall quality of evidence from non-randomised clinical trials began with low quality. In general, the outcome of PI, BOP, and PPD was determined as having very low quality due to the involvement of non-randomised clinical trials, imprecision, and a high risk of bias within the studies. The quality pertaining to the GI was evaluated as low due to involving non-randomised clinical trials.

**Table 5:** Summary of findings table according to the GRADE approach

Comparative periodontal status between fixed labial and fixed lingual orthodontic treatment				
Patient or population: patients receiving fixed labial or lingual orthodontic treatment Intervention: Settings: Periodontal status between fixed labial and lingual orthodontic treatment				
Outcomes	Relative effect* (95% CI)	No. of participants (studies)	Quality of evidence (GRADE)	Comments
	Corresponding risk Periodontal status between fixed labial and lingual orthodontic treatment			
Plaque index	The mean plaque index in the intervention groups was 0.14 lower (0.27 to 0.02 lower)	220 (3 studies)	⊕⊖⊖⊖ very low^1,2^	The plaque index was lower in the fixed lingual orthodontic therapy compared with the fixed labial orthodontic treatment.
Periodontal pocket depth	The mean periodontal pocket depth in the intervention groups was 0.06 higher (0.16 lower to 0.27 higher)	260 (3 studies)	⊕⊖⊖⊖ very low^1,2,3,4^	No statistically significant difference was found in periodontal pocket depth between the fixed labial and lingual orthodontic treatment.
Bleeding on probing index	The mean bleeding on probing index in the intervention groups was 0.11 higher (0.03 lower to 0.25 higher)	180 (2 studies)	⊕⊖⊖⊖ very low^1,2,3^	No statistically significant difference was found in bleeding on probing index between the fixed labial and lingual orthodontic treatment.
Gingival index	The mean gingival index in the intervention groups was 0.02 higher (0.06 lower to 0.11 higher)	280 (4 studies)	⊕⊕⊖⊖ low^1,2,3^	No statistically significant difference was found in gingival index between the fixed labial and lingual orthodontic treatment.

*The basis for the assumed risk (e.g. the median control group risk across studies) is provided in footnotes. The corresponding risk (and its 95% confidence interval (CI]) is based on the assumed risk in the comparison group and the relative effect of the intervention (and its 95% CI).

GRADE Working Group grades of evidence High quality: Further research is very unlikely to change our confidence in the estimate of effect. Moderate quality: Further research is likely to have an important impact on our confidence in the estimate of effect and may change the estimate. Low quality: Further research is very likely to have an important impact on our confidence in the estimate of effect and is likely to change the estimate. Very low quality: We are very uncertain about the estimate.

^1^ Non-randomised clinical trials invovled. ^2^ The sample size of the meta-analysis was limited to draw a precise estimate of effect. ^3^ There was at least one study that was assessed as having high or serious risk of bias. ^4^ There was high heterogeneity across the involved studies.

## Discussion

Maintaining an acceptable level of oral hygiene was of great importance during the course of orthodontic treatment to avoid any adverse effects related to periodontal health. The current review systematically examined all available evidence on clinical periodontal parameters during the course of fixed labial and lingual orthodontic treatment. Based on the 8 included clinical investigations with 223 patients, the current review found no statistically significant differences in periodontal parameters between fixed labial and lingual orthodontic systems. However, the overall quality was very low to low, indicating that further RCTs with larger sample size and standardised outcome measures are needed.

In the past, it was generally acknowledged that patients with fixed lingual orthodontic appliances should pay more attention to oral hygiene, as it was more difficult to conduct daily oral hygiene procedures blindly from the lingual side, also given the wider brackets and reduced inter-bracket distance.^14,15^ However, our results found a lower plaque index in the lingual group; this could partly be due to the lingual surface of the teeth being closer to the tongue and the open salivary duct. It has been reported that the flushing effect of saliva might interfere with bacterial adherence and biofilm formation on the lingual side.^7,9,21^ Moreover, the activity of the tongue also contributes to the mechanical cleaning of dental plaque on the lingual side of the teeth.^39^ These two self-cleaning mechanisms might help reduce plaque accumulation on the lingual surface of the teeth.^39^ However, this finding should be interpreted with great caution, due to the very low quality of evidence. No statistically significant difference was found concerning the BOP, GI, and PPD between patients receiving the fixed labial and those who received lingual orthodontic treatment. The results of BOP, GI, and PPD were inconsistent with the results of PI according to our study. A possible explanation might be that the follow-up duration of most of the included investigations was too short to detect biological alterations in the periodontal tissues.^38^ Therefore, further investigations with longer follow-ups are warranted to confirm the results of the current study. In addition, since gingivitis is a risk factor for attachment loss and clinical attachment loss (CAL), which has been highlighted as one of the characteristics of periodontitis,^37^ future studies focusing on differences in attachment loss between patients receiving fixed labial vs lingual orthodontic treatment should be conducted.

Clinically, treatment outcome and safety in terms of periodontal health were not the sole considerations when patients chose the appliance type and therapeutic strategy for orthodontic treatment.^25^ The cost was also an important influencing factor, as lingual orthodontics is currently considerably more expensive due to its manufacturing technology. Therefore, patients should be instructed about treatment effects, potential adverse effects, and costs when choosing orthodontic appliances to ensure that patient is well-informed about treatment planning.

### Strengths and Limitations

Previous reviews focused on the self-reported difficulty in conducting daily oral hygiene measures.^1,27^ The patient-report outcome could not reflect the real clinical periodontal status. The present study was the first systematic review to consider all available evidence using objective clinical periodontal parameters to compare periodontal health between patients receiving fixed labial and lingual orthodontic treatment. Our study thus constitutes a comprehensive and systematic evaluation of the efficacy of contemporary fixed orthodontic appliances in helping maintain periodontal health during treatment.

However, there are some limitations to the current systematic review and meta-analysis, the main one being the inclusion of non-randomised clinical trials.^2^ Although randomised controlled clinical trials are considered the gold standard for clinical trials, performing random assignment requires extra financial support. The fact that the selection of orthodontic appliances is influenced by the patient’s socioeconomic status and their aesthetic requirements must also be considered.^17^ Similarly, allocation concealment is also difficult to perform due to the nature and appearance of the orthodontic appliances. Thus, non-randomised clinical trials were also considered acceptable. Moreover, in the current study, the limited number of investigations included prohibited robust assessments of heterogeneity, subgroup analyses for many confounding factors, and publication bias assessment.^28^ Additionally, the sample size of the included studies was small, which leads to the retrieved results lacking adequate precision. Also the short follow-up duration in most included studies precludes the assessment of the long-term influence of the orthodontic appliance on periodontal health, since such biological alterations take time. Therefore, more studies comparing the clinical periodontal parameters between the fixed labial and lingual orthodontic technique with a larger sample size, standardised methodology, and longer follow-up period are recommended to confirm the conclusions of the present review.

## Conclusions

This review found that the periodontal status in patients undergoing fixed labial and lingual orthodontic treatment was similar. From the clinical point of view, the fixed lingual orthodontic appliance is comparable to the fixed labial orthodontic appliance with regard to the clinical periodontal parameters. However, more studies with high quality, larger sample size, and longer follow-up are necessary in order to validate our findings.
